# Epidermal growth factor receptor and notch signaling in non‐small‐cell lung cancer

**DOI:** 10.1002/cam4.944

**Published:** 2016-10-21

**Authors:** Joanna Pancewicz‐Wojtkiewicz

**Affiliations:** ^1^ Department of Histology and EmbryologyMedical University of BialystokBialystokPoland

**Keywords:** Epidermal growth factor receptor, non‐small‐cell lung cancer, Notch, tyrosine kinase inhibitors

## Abstract

Lung cancer is the most common reason of cancer deaths and about 85% of these are non‐small‐cell lung cancer. Currently, lung cancer therapy is mainly based on the tumor node metastasis (TNM) disease staging and tumor histological classification. Despite therapeutic innovations, the prognosis for lung cancer patients has not significantly changed in the last years. Therefore, a proper understanding of cell signaling pathways involved in cancer pathogenesis seems to be essential for improvement in cancer therapy field. The knowledge of crosstalk between epidermal growth factor receptor (EGFR) and Notch pathway can lead to enhanced screening for the expression of these genes allowing patients to optimize treatment options and predict potential treatment resistance. This review focuses on recent advances related to the mechanisms of EGFR and Notch signaling in non‐small‐cell lung cancer and the effectiveness of current Notch‐ and EGFR‐targeted therapies.

## Introduction

### EGFR alterations in non‐small‐cell lung cancer in brief

Epidermal growth factor receptor (EGFR) has tyrosine kinase activity and is a transmembrane glycoprotein. The EGFR and members of its family play a significant role in carcinogenesis through contribution into cell proliferation, apoptosis, cell motility, and angiogenesis. EGFR alterations are involved in the pathogenesis and progression of many malignancies including lung cancer 1, 2, 3, 4, 5.

One of the most common alteration in non‐small‐cell lung cancer (NSCLC) patients and cells is overexpression of EGFR, which is demonstrated in more than half cases of NSCLC and is associated with a poor prognosis and chemoresistance 6, 7. Moreover, the expression of EGFR appears to be dependent on histological subtypes of NSCLC, and is most frequently expressed in squamous cell. In addition to EGFR overexpression, activating mutations of EGFR are observed in around 10% of all nonsquamous non‐small‐cell lung cancer patients 8.

Epidermal growth factor receptor mutations are significant predictors of treatment response to tyrosine kinase inhibitors (TKISs) in patients with non‐small‐cell lung cancer. However, according to researchers, diverse response to the treatment is common. Therefore, there are group of patients with mutations who do not show any response and some patients without mutations who can respond to the treatment. Moreover, other investigators 9 discovered additional alterations of EGFR in NSCLC patients (Table 1). Winter‐Larsen et al. identified genetic polymorphism of the EGFR gene and expected it may be important for prediction of clinical consequences in TKISs‐treated advanced NSCLC patients 10. Furthermore, increased EGFR copy numbers were described as a common modification in NSCLC. Altered EGFR copy numbers are present up to 59% of NSCLC 11, 12, 13, 14. According to Sholl et al. and other research groups 14, 16, gain of EGFR copy number is related to a positive effect after EGFR TKISs treatment; it has also been proposed to be a potential biomarker of TKISs responsiveness. Likewise, treatment with TKISs gives better results in positive EGFR samples 12, 15. In addition to described alteration, EGFR methylation and phosphorylation might have strong impact on the clinical outcome of NSCLC. Thus, Li et al. discovered the importance of EGFR gene methylation, which was associated with malignancy of this type of cancer 17. In other study, patients with phospho‐EGFR‐positive tumors demonstrated a longer survival 18. On the other hand, Hijiya et al. investigated 21 cases of NSCLC to examine correlations between the existence of EGFR mutations and the EGFR phosphorylation grade by immunohistochemistry. Moreover, the mutation status of the EGFR gene was correlated with immunoreactivity for phosphor‐EGFR and its immunoreactivity was significantly correlated with clinical responsiveness to one of the available drug—gefitinib 19. Taking together, the alterations of EGFR are common condition in NSCLC patients and usually correlate with poor prognosis and resistance to chemotherapy.

**Table 1:** Aberrations in EGFR and Notch signaling pathways in NSCLC

Pathway	Alteration	Clinical implication	Source
EGFR	Overexpression	Poor prognosis, chemoresistance	6, 7
	Mutations	Predictors to TKISs response	8
	Copy number variations	Potential biomarker of TKISs responsiveness	11, 12, 13, 14
	Methylation status	Positive correlation with malignancy	17
	Phosphorylation status	Survival predictor	18
Notch	Dysregulated expression	Poor prognosis	24, 25, 26, 27
	Mutations	Poor prognosis	28

EGFR, epidermal growth factor receptor; NSCLC, non‐small‐cell lung cancer; TKISs, tyrosine kinase inhibitors.

### Notch in non‐small‐cell lung cancer in brief

**Figure 1:**
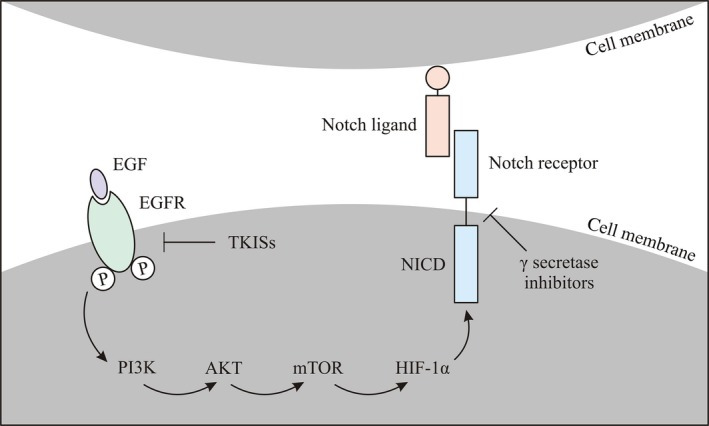
The scheme shows that activation of EGFR triggers PI3K/AKT/mTOR pathways cascade, which increases the translation of hypoxia‐inducible factors. Moreover, hypoxia can stabilize NICD by interaction with HIF‐1*α*. TKISs, tyrosine kinase inhibitors; EGF, epidermal growth factor; EGFR, epidermal growth factor receptor; PI3K/AKT, phosphatidylinositide 3‐kinases; mTOR, mechanistic target of rapamycin; NICD, intracellular domain of Notch; HIF‐1*α*, hypoxia‐inducible factors.

The Notch signaling pathway is conservative and plays an important role in the cellular proliferation, differentiation, and apoptosis. The human Notch family includes four receptors (Notch 1 through 4 in mammalians) and five ligands (Jagged1, Jagged2, Dll1, Dll3, and Dll4) 20, 21. Activation of Notch pathway depends on interaction between specific ligand and receptor; nevertheless different mechanisms are involved in this process. The canonical way occurs when NICD is released after enzymatic intervention of ADAM family metalloprotease, which creates a substrate for a second cleavage by the *γ*‐secretase complex, releasing the Notch intracellular domain (NICD). The intracellular domain is later moved into the nucleus where it cooperates with CBF‐1 (transcription factor recombining binding protein suppressor of hairless). The noncanonical way can take place without *γ*‐secretase cleavage and CBF‐1 20. Although, the mechanisms of Notch activation are known in physiological conditions, the processes regulating this pathway in cancer are not so evident. It has been postulated that hypoxic tumor microenvironment may be crucial in regulation of Notch pathway in cancer. Moreover, evaluation of Notch pathway expression in cancer may not be related only to up‐ or downregulation of this signaling, but may be determined by compound interactions with EGFR through activation of PI3K/AKT/mTOR cascade which in turn increases the translation of hypoxia inducible factors (HIF‐1*α*). Therefore, according to some authors, hypoxia stabilizes NICD which can interact with hypoxia‐inducible factor 1 alpha (HIF‐1*α*) (Fig. 1) 22, 23.

There are some studies indicating Notch is highly activated in NSCLC 24, 25. However, other studies demonstrated a reduced or undetectable Notch1 expression in NSCLC. This implies a supposed Notch1 tumor‐suppressive role in these tumors and again gives a notion that Notch function in NSCLC is more complicated than predicted 26, 27.

On the other hand, altered Notch genes may influence the stabilization of Notch in NSCLC. Hence, two types of alterations were detected in NSCLC: heterozygous mutations of the Notch‐1 locus in 10% of the cases, and loss of Numb expression in 30% of the cases of NSCLC 28.

Although, the role of Notch in non‐small‐cell lung cancer remains unclear, the observations reveal that Notch signaling in NSCLC depends on the specific tissue context, microenvironment, and crosstalk with other signaling pathways. Consequently, it might be important in development of tumor or can act as a tumor suppressor 24. Although the mechanism of Notch signaling in lung cancer pathogenesis is not fully understood, most likely other factors are involved.

### Clinical relevance and therapeutic approaches aimed at targeting Notch and EGFR signaling in NSCLC

Many research groups try to explain crosstalk between Notch and EGFR in order to understand the mechanism of this cooperation and to know how cancer cells use the Notch pathway to compensate for EGFR‐targeted inhibition. Notch and epidermal growth factor receptor (EGFR) signaling are essential in cell proliferation, differentiation, and apoptosis, and thereby may contribute to the development of lung cancer.

**Figure 2:**
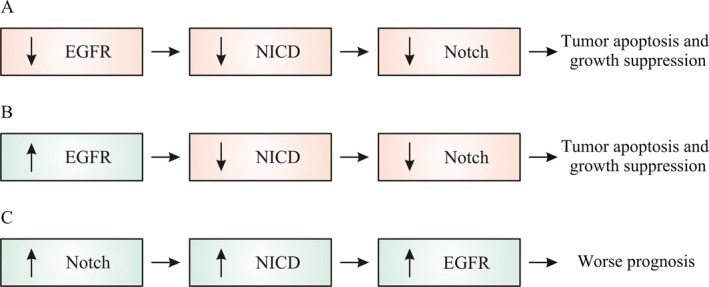
Possible mechanisms of crosstalk between EGFR and Notch and clinical consequences: (A) EGFR cooperates with intracellular domain of Notch by enhancing its effect on tumor apoptosis, (B) EGFR overexpression downregulates Notch, (C) Notch upregulates EGFR expression through p53 as a mediator of the Notch‐1. NICD, intracellular domain of Notch.

It has been described that those pathways can cooperate in different mechanisms, either antagonistic or synergistic (Fig. 2), depending on tissue, developmental status, and microenvironment 29.

Although recent studies have shown that Notch and EGFR signaling are associated with drug resistance, antiangiogenic agent and EGFR tyrosine kinase inhibitors have been accepted for NSCLC treatment 30, 31.

Moreover, current clinical trials examine the efficacy and safety of antiangiogenic and anti‐EGFR agents combinations, as well as additional agents such as multitargeted antiangiogenic tyrosine kinase inhibitors 32, 33.

The researchers revealed that the expression of Notch‐1 was upregulated in EGFR‐TKISs developed resistant lung cancer cells. Additional, Notch‐1 contributed to the achievement of the epithelial–mesenchymal transition (EMT) phenotype, which was correlated with developed resistance to EGFR‐TKISs 34.

Another study showed that while inhibition of EGFR leads to reduction in tumor cell number, it also leads to a potent activation of the Notch pathway. Combined inhibition of EGFR and Notch3 receptors significantly reduced the growth of stem‐like cells. Taking together, investigators concluded that treatment of EGFR‐mutated lung cancer cell lines with erlotinib enriched then stem‐like cells with stem‐like cell potential through EGFR‐dependent activation of Notch3. Moreover, *γ* ‐secretase inhibitors could reverse this phenotype. Furthermore, the scientists noticed that phosphorylation of Notch3 can be linked to EGFR receptor, but no exact mechanism is known yet 35.

The crosstalk between Notch and EGFR pathway was also conducted by Konishi et al. and Kolev et al. The investigators demonstrated that the interaction between both pathways results in the inhibition of apoptosis 36, 37. Although independent results presented in gliomas indicated that Notch may upregulates EGFR through p53 38, another study showed that inhibition of Notch cleavage may not change cell number in the presence of EGFR mutations. Moreover, EGFR may affect Notch signaling suggesting that inhibition of both pathways could be promising in NSCLC. The researchers selected four NSCLC cell lines expressing different levels of NICD (intracellular domain of Notch) and EGFR protein levels and found that the cell lines exhibited different response to the *γ*‐secretase inhibitor DAPT (N‐[N‐(3,5‐difluorophenacetyl)‐l‐alanyl]‐S‐phenylglycine t‐butyl ester) and related this to EGFR status. DAPT was effective in proliferation of cells expressing wt EGFR (wild type), whereas it did not affect HCC827 cells expressing mutated EGFR. In addition, alterations were observed among the cells with wild‐type EGFR 39. Another groups of investigators focused on EGFR and Notch ligands. Correspondingly, Choi et al. examined Jag1 expression regulated by EGFR. Nevertheless, Jag2, which belongs to the same group of ligands, was not regulated by EGFR. To examine the role of EGFR using a different approach, wild‐type EGFR H1299 cells, which indicated low levels of Jag1 and Jag2 expression, were treated with EGF or transfected with wild‐type EGFR. As a result, two of the transfected agents increased only the expression of Jag1 and gefitinib treatment abolished EGFR‐induced Jag1 expression in H1299 cells 40. The discovery of EGFR mutations in non‐small‐cell lung cancer initiated the personalized medicine in advanced NSCLC. During the last decade, different EGFR‐TKISs have been developed. Three EGFR inhibitors, gefitinib, erlotinib, and afatinib, are already used in treatment for patients with NSCLC (Tables 2 and 3). Nevertheless, despite great advances have been made, novel treatment still should overcome the therapeutic challenges, such as resistance or metastases 41.

**Table 2:** The most promising Notch and EGFR inhibitors list for targeted therapy of NSCLC

	Targets
Notch inhibitors	
neutralizing monoclonal antibodies: OMP‐59R5, OMP‐21M18, NRR1, NRR2	Notch receptors and ligands
*γ*‐secretase inhibitors: RO4929097, MRK‐0752, PF‐03084014, MRK‐003, BMS‐906024	Blocking proteolytic activation of Notch receptors
EGFR inhibitors	
erlotinib, afatinib, gefitinib	EGFR gene mutations
osimertinib, rociletinib, dacomitinib	Cells with the T790M mutation
anti‐EGFR monoclonal antibodies: cetuximab, nimotuzumab, panitumumab	Three agents act on the same target (EGFR)

EGFR, epidermal growth factor receptor; NSCLC, non‐small‐cell lung cancer.

**Table 3:** Effectiveness of Notch‐ and EGFR‐targeted therapies in NSCLC

	Effectiveness of current Notch‐ and EGFR‐targeted therapy in NSCLC	References
Notch‐targeted therapies		
Inhibition of Notch signaling with available *γ*‐secretase inhibitors, mAbs, arsenic trioxide (animal model)	Affect tumor cells survival, differentiation, angiogenesis; drawbacks—toxicity	42, 43, 44
EGFR‐targeted therapies		
Inhibition of mutated EGFR with TKISs inhibitors	Efficient in NSCLC patients with mutated EGFR, effectiveness in the treatment of brain metastases from NSCLC; drawbacks—cancer cells develop new mutations in the EGFR gene	45, 46, 47
Inhibition of mutated EGFR with mAbs	Used with chemotherapy as the first treatment in people with advanced squamous cell NSCLC inhibit tumor growth	48, 49
Combined Notch‐/EGFR‐targeted therapies		
A phase I/II trial combining erlotinib (E) with *γ*‐secretase inhibitor RO4929097(R) in advanced NSCLC	Combination of R and E is safe in patients with NSCLC; clinical trial information: NCT01193881	50
Combined Notch/EGFR therapy with *γ*‐secretase inhibitor (DAPT) N‐[N‐(3,5‐difluorophenacetyl)‐l‐alanyl]‐(S)‐phenylglycine t‐butyl ester and gefitinib (animal model)	Effective tumor growth inhibition, with decreased proliferative activity and increased apoptotic activity	34

mAbs, monoclonal antibodies; E, erlotinib; R, *γ*‐secretase inhibitor RO4929097; TKISs, tyrosine kinase inhibitors; DAPT, N‐[N‐(3,5‐difluorophenacetyl)‐L‐alanyl]‐(S)‐phenylglycine t‐butyl ester.

## Conclusions

As researchers have developed knowledge about the alterations in lung cancer cells that help them grow, they have developed newer drugs that specifically target these changes. Despite of new drugs and therapeutic regiments, the prognosis for lung cancer patients has not significantly transformed in the last years. There is now overwhelming data on the prognostic and predictive value of each EGFR signaling in NSCLC. Although the role of EGFR signaling in the pathogenesis and progression of NSCLC is well recognized, the importance of Notch pathway and its correlation with EGFR in lung cancer is still under investigation. Notch may act as an oncogene or a tumor suppressor gene in lung cancer cells depending on tissue, developmental context, and microenvironment. However, recently agents targeting the fundamental molecular signaling pathways in lung cancer are already under clinical trials with more promising results. Thus, the mechanism(s) of crosstalk between EGFR and Notch in non‐small‐cell lung cancer need to be identified.

## Conflict of Interest

The author declares that there is no conflict of interests regarding the publication of this study.

## References

[1] Ciardiello, F. , and G. Tortora . 2008 EGFR antagonists in cancer treatment. N. Engl. J. Med. 358:1160–1174. 18337605 10.1056/NEJMra0707704

[2] Ciardiello, F. , and G. Tortora . 1998 Interactions between the epidermal growth factor receptor and type I protein kinase A: biological significance and therapeutic implications. Clin. Cancer Res. 4:821–828. 9563874

[3] Lynch, T. J., D. W. Bell , R. Sordella , S. Gurubhagavatula , R. A. Okimoto , B. W. Brannigan , et al. 2004 Activating mutations in the epidermal growth factor receptor underlying responsiveness of non‐small‐cell lung cancer to gefitinib. N. Engl. J. Med. 350:2129–2139. 15118073 10.1056/NEJMoa040938

[4] Pao, W. , and V. A. Miller . 2005 Epidermal growth factor receptor mutations, small‐molecule kinase inhibitors, and non‐small‐cell lung cancer: current knowledge and future directions. J. Clin. Oncol. 23:556–2568. 10.1200/JCO.2005.07.799 15767641

[5] Cheng, L. , and D. Zhang . 2008 Molecular genetic pathology. Humana Press/Springer, New York, NY.

[6] Reade, C. A. , and A. K. Ganti . 2009 EGFR targeted therapy in non‐small cell lung cancer: potential role of cetuximab. Biogeosciences 3:215–224. PMC2726075 19707410

[7] Cheng, L. , S. Zhang , R. Alexander , Y. Yao , G. T. MacLennan , C. X. Pan , et al. 2011 The landscape of EGFR pathways and personalized management of non‐small‐cell lung cancer. Future Oncol. 7:519–541. 21463141 10.2217/fon.11.25

[8] Lopes, G. L. , E. F. Vattimo , and G. de Castro Junior . 2015 Identifying activating mutations in the EGFR gene: prognostic and therapeutic implications in non‐small cell lung cancer. J Bras. Pneumol. 41:365–375. 26398757 10.1590/S1806-37132015000004531 PMC4635957

[9] Tsiambas, E. , V. Ragos , A. Y. Lefas , S. N. Georgiannos , D. Grapsa , E. Patsouris , et al. 2015 Chromosome 7 deregulation in non‐small cell lung carcinoma molecular landscape. J BUON 20:1635–1639. 26854464

[10] Winther‐Larsen, A. , P. H. Nissen , K. R. Jakobsen , C. Demuth , B. S. Sorensen , and P. Meldgaard . 2015 Genetic polymorphism in the epidermal growth factor receptor gene predicts outcome in advanced non‐small cell lung cancer patients treated with erlotinib. Lung Cancer 90:314–320. 26386832 10.1016/j.lungcan.2015.09.003

[11] Dahabreh, I. J. , H. Linardou , F. Siannis , P. Kosmidis , D. Bafaloukos , and S. Murray . 2010 Somatic EGFR mutation and gene copy gain as predictive biomarkers for response to tyrosine kinase inhibitors in non‐small cell lung cancer. Clin. Cancer Res. 16:291–303. 20028749 10.1158/1078-0432.CCR-09-1660

[12] Hirsch, F. R. , R. S. Herbst , C. Olsen , K. Chansky , J. Crowley , K. Kelly , et al. 2008 Increased EGFR gene copy number detected by fluorescent in situ hybridization predicts outcome in non‐small‐cell lung cancer patients treated with cetuximab and chemotherapy. J. Clin. Oncol. 26:3351–3357. 18612151 10.1200/JCO.2007.14.0111 PMC3368372

[13] Cappuzzo F. , F. R. Hirsch , E. Rossi , S. Bartolini , G. L. Ceresoli , L. Bemis , et al. 2005 Epidermal growth factor receptor gene and protein and gefitinib sensitivity in non‐small‐cell lung cancer. J. Natl Cancer Inst. 97:643–655. 15870435 10.1093/jnci/dji112

[14] Sholl, L. M. , A. John Iafrate , Y. P. Chou , M. T. Wu , Y. G. Goan , L. Su , et al. 2007 Validation of chromogenic in situ hybridization for detection of EGFR copy number amplification in nonsmall cell lung carcinoma. Mod. Pathol. 20:1028–1035. 17673923 10.1038/modpathol.3800946

[15] Tsao, M. S. , A. Sakurada , J. C. Cutz , C. Q. Zhu , S. Kamel‐Reid , J. Squire , et al. 2005 Erlotinib in lung cancer – molecular and clinical predictors of outcome. N. Engl. J. Med. 353:133–144. 16014883 10.1056/NEJMoa050736

[16] Cappuzzo, F. , C. Ligorio , P. A. Jänne , L. Toschi , E. Rossi , R. Trisolini , et al. 2007 Prospective study of gefitinib in epidermal growth factor receptor fluorescence in situ hybridization‐positive/phospho‐AKT‐positive or never smoker patients with advanced non‐small‐cell lung cancer: the ONCOBELL trial. J. Clin. Oncol. 25:2248–2255. 17538169 10.1200/JCO.2006.09.4300

[17] Li, J. , X. F. Jia , J. Liu , J. J. Liu , and H. B. Zhao . 2015 Relationship of EGFR DNA methylation with the severity of non‐small cell lung cancer. Genet. Mol. Res. 14:11915–11923. 26505339 10.4238/2015.October.5.5

[18] Endoh, H. , Y. Ishibashi , E. Yamaki , T. Yoshida , T. Yajima , H. Kimura , et al. 2009 Immunohistochemical analysis of phosphorylated epidermal growth factor receptor might provide a surrogate marker of EGFR mutation. Lung Cancer 63:241–246. 18585821 10.1016/j.lungcan.2008.05.013

[19] Hijiya, N. , M. Miyawaki , K. Kawahara , S. Akamine , K. Tsuji , J. Kadota , et al. 2008 Phosphorylation status of epidermal growth factor receptor is closely associated with responsiveness to gefitinib in pulmonary adenocarcinoma. Hum. Pathol. 39:316–323. 18261621 10.1016/j.humpath.2007.10.027

[20] Pancewicz, J. , and C. Nicot . 2011 Current views on the role of Notch signaling and the pathogenesis of human leukemia. BMC Cancer 11:502. 22128846 10.1186/1471-2407-11-502 PMC3262490

[21] Liu, N. , J. Zhang , and C. Ji . 2013 Ankyrin/CDC10 repeats (ANK), necessary for protein‐protein interactions. Biomark. Res. 1:23.24252593

[22] Andersson, E. R. , R. Sandberg , and U. Lendahl . 2011 Notch signaling: simplicity in design, versatility in function. Development 138:3593–3612. 21828089 10.1242/dev.063610

[23] Yamaguchi, H. , S. S. Chang , J. L. Hsu , and M. C. Hung . 2014 Signaling cross‐talk in the resistance to HER family receptor targeted therapy. Oncogene 33:1073–1081. 23542173 10.1038/onc.2013.74 PMC3874419

[24] Brizzi, M. F. , and P. Defilippi . 2013 Dll4/Notch1 signaling from tip/stalk endothelial cell specification to stroma‐dependent lung tumor inhibition: a flavor of Dll4/Notch1 pleiotropy in tumor cell biology. Transl. Lung Cancer Res. 2:466–469. 25806273 10.3978/j.issn.2218-6751.2013.10.18 PMC4367631

[25] Yuan, X. , H. Wu , H. Xu , N. Han , Q. Chu , S. Yu , et al. 2015 Meta‐analysis reveals the correlation of Notch signaling with non‐small cell lung cancer progression and prognosis. Sci. Rep. 5:10338. 25996086 10.1038/srep10338 PMC4440529

[26] Chen, Y. , M. A. De Marco , I. Graziani , A. F. Gazdar , P. R. Strack , L. Miele , et al. 2007 Oxygen concentration determines the biological effects of NOTCH‐1 signaling in adenocarcinoma of the lung. Cancer Res. 67:7954–7959. 17804701 10.1158/0008-5472.CAN-07-1229

[27] Konishi, J. , K. S. Kawaguchi , H. Vo , N. Haruki , A. Gonzalez , D. P. Carbone , et al. 2007 Gamma‐secretase inhibitor prevents Notch3 activation and reduces proliferation in human lung cancers. Cancer Res. 67:8051–8057. 17804716 10.1158/0008-5472.CAN-07-1022

[28] Westhoff, B. , I. N. Colaluca , G. D'Ario , M. Donzelli , D. Tosoni , S. Volorio , et al. 2009 Alterations of the Notch pathway in lung cancer. Proc. Natl Acad. Sci. USA 106:22293–22298. 20007775 10.1073/pnas.0907781106 PMC2799768

[29] Doroquez, D. B. , and I. Rebay . 2006 Signal integration during development: mechanisms of EGFR and Notch pathway function and cross‐talk. Crit. Rev. Biochem. Mol. Biol. 41:339–385. 17092823 10.1080/10409230600914344

[30] Aita, M. , G. Fasola , C. Defferrari , A. Brianti , M. G. Bello , A. Follador , et al. 2008 Targeting the VEGF pathway: antiangiogenic strategies in the treatment of non‐small cell lung cancer. Crit. Rev. Oncol. Hematol. 68:183–196. 18606548 10.1016/j.critrevonc.2008.05.002

[31] Adamo, V. , T. Franchina , B. Adamo , N. Denaro , P. Gambadauro , G. Chiofalo , et al. 2009 Gefitinib in lung cancer therapy: clinical results, predictive markers of response and future perspectives. Cancer Biol. Ther. 8:206–212. 19182534 10.4161/cbt.8.3.7465

[32] Scagliotti, G. , and R. Govindan . 2010 Targeting angiogenesis with multitargeted tyrosine kinase inhibitors in the treatment of non‐small cell lung cancer. Oncologist 15:436–446. 20427383 10.1634/theoncologist.2009-0225 PMC3227980

[33] Pennell, N. A. , and T. J. Jr Lynch . 2009 Combined inhibition of the VEGFR and EGFR signaling pathways in the treatment of NSCLC. Oncologist 14:399–411. 19357226 10.1634/theoncologist.2008-0276

[34] Xie, M. , C. S. He , S. H. Wei , and L. Zhang . 2013 Notch‐1 contributes to epidermal growth factor receptor tyrosine kinase inhibitor acquired resistance in non‐small cell lung cancer in vitro and in vivo. Eur. J. Cancer 49:3559–3572. 23916913 10.1016/j.ejca.2013.07.007

[35] Arasada, R. R. , J. M. Amann , M. A. Rahman , S. S. Huppert , and D. P. Carbone . 2014 EGFR blockade enriches for lung cancer stem‐like cells through Notch3‐dependent signaling. Cancer Res. 74:5572–5584. 25125655 10.1158/0008-5472.CAN-13-3724 PMC4263272

[36] Konishi, J. , F. Yi , X. Chen , H. Vo , D. P. Carbone , and T. P. Dang . 2010 Notch3 cooperates with the EGFR pathway to modulate apoptosis through the induction of bim. Oncogene 29:589–596. 19881544 10.1038/onc.2009.366 PMC2813325

[37] Kolev, V. , A. Mandinova , J. Guinea‐Viniegra , B. Hu , K. Lefort , C. Lambertini , et al. 2008 EGFR signaling as a negative regulator of Notch1 gene transcription and function in proliferating keratinocytes and cancer. Nat. Cell Biol. 10:902–911. 18604200 10.1038/ncb1750 PMC2747621

[38] Purow, B. W. , T. K. Sundaresan , M. J. Burdick , B. A. Kefas , L. D. Comeau , M. P. Hawkinson , et al. 2008 Notch‐1 regulates transcription of the epidermal growth factor receptor through p53. Carcinogenesis 29:918–925. 18359760 10.1093/carcin/bgn079 PMC2902388

[39] Giannopoulou, E. , A. Nikolakopoulos , D. Kotsirilou , A. Lampropoulou , S. Raftopoulou , E. Papadimitriou , et al. 2015 Epidermal growth factor receptor status and Notch inhibition in non‐small cell lung cancer cells. J. Biomed. Sci. 22:98. 26497899 10.1186/s12929-015-0196-1 PMC4619334

[40] Choi, K. , Y. H. Ahn , D. L. Gibbons , H. T. Tran , C. J. Creighton , L. Girard , et al. 2009 Distinct biological roles for the Notch ligands Jagged‐1 and Jagged‐2. J. Biol. Chem. 284:17766–17774. 19398556 10.1074/jbc.M109.003111 PMC2719415

[41] Russo, A. , T. Franchina , G. R. Ricciardi , A. Picone , G. Ferraro , M. Zanghì , et al. 2015 A decade of EGFR inhibition in EGFR‐mutated non‐ small cell lung cancer (NSCLC): old successes and future perspectives. Oncotarget 6:26814–26825. 26308162 10.18632/oncotarget.4254 PMC4694955

[42] Purow, B. 2012 Notch inhibition as a promising new approach to cancer therapy. Adv. Exp. Med. Biol. 727:305–319. 22399357 10.1007/978-1-4614-0899-4_23 PMC3361718

[43] Luistro, L. , W. He , M. Smith , K. Packman , M. Vilenchik , D. Carvajal , et al. 2009 Preclinical profile of a potent gamma‐secretase inhibitor targeting notch signaling with in vivo efficacy and pharmacodynamic properties. Cancer Res. 69:7672–7680. 19773430 10.1158/0008-5472.CAN-09-1843 PMC5260798

[44] Yang, M. H. , Y. S. Zang , H. Huang , K. Chen , B. Li , G. Y. Sun , et al. 2014 Arsenic trioxide exerts anti‐lung cancer activity by inhibiting angiogenesis. Curr. Cancer Drug Targets 14:557–566. 25088040 10.2174/1568009614666140725090000

[45] Chonan, M. , N. Narita , and T. Tominaga . 2016 Total regression of brain metastases in non‐small cell lung cancer patients harboring EGFR mutations treated with gefitinib without radiotherapy: two case reports. BMC Res. Notes 9:2. 26724810 10.1186/s13104-015-1834-0 PMC4698324

[46] Protsenko, S. A. , and A. V. Rudakova . 2015 Gefitinib therapy in advanced non‐small cell lung cancer in patients with EGFR mutations: cost‐effectiveness analysis. Vopr. Onkol. 61:676–680. 26571844

[47] Liu, L. L. , F. Li , W. Pao , and F. Michor . 2015 Dose‐dependent mutation rates determine optimum erlotinib dosing strategies for EGFR mutant non‐small cell lung cancer patients. PLoS ONE 10:11. 10.1371/journal.pone.0141665 PMC4633116 26536620

[48] Bhardwaj, B. , S. Revannasiddaiah , H. Bhardwaj , S. Balusu , and A. Shwaiki . 2016 Molecular targeted therapy to improve radiotherapeutic outcomes for non‐small cell lung carcinoma. Ann. Transl. Med. 4:50. 26904572 10.3978/j.issn.2305-5839.2015.10.35 PMC4740001

[49] Hu, Y. , X. Z. Dong , X. Liu , P. Liu , and Y. B. Chen . 2016 Enhanced antitumor activity of cetuximab in combination with the jak inhibitor CYT387 against non‐small‐cell lung cancer with various genotypes. Mol. Pharm. 13:689–697. 26685983 10.1021/acs.molpharmaceut.5b00927

[50] Gold, K. A. , L. A. Byers , Y. H. Fan , J. Fujimoto , W. H. Tse , J. Jack Lee , et al. 2013 A phase I/II trial combining erlotinib with gamma secretase inhibitor RO4929097 in advanced non‐small cell lung cancer (NSCLC). J. Clin. Oncol. 31(Suppl): abstr 8104.

